# Chromosomal Aberrations in Lymphocytes of Healthy Subjects and Risk of Cancer

**DOI:** 10.1289/ehp.6925

**Published:** 2005-02-02

**Authors:** Pavel Rossner, Paolo Boffetta, Marcello Ceppi, Stefano Bonassi, Zdenek Smerhovsky, Karel Landa, Dagmar Juzova, Radim J. Šrám

**Affiliations:** ^1^Department of Genetic Ecotoxicology, Institute of Experimental Medicine, Academy of Sciences of the Czech Republic, and Health Institute of Central Bohemia, Prague, Czech Republic;; ^2^International Agency for Research on Cancer, Lyon, France;; ^3^Epidemiology and Biostatistics, National Cancer Research Institute, Genova, Italy;; ^4^Center of Industrial Hygiene and Occupational Diseases, National Institute of Public Health, Prague, Czech Republic

**Keywords:** cancer risk, chromosomal aberrations, cohort study, cytogenetic assay, molecular epidemiology

## Abstract

There is evidence that increased frequency of chromosomal aberration (CA) in peripheral blood lymphocytes is a predictor of cancer, but further data are needed to better characterize CA as marker of cancer risk. From the archives of 15 laboratories we gathered cytogenetic records of 11,834 subjects who were free of cancer at the moment of blood drawing and who underwent cytogenetic examination for preventive purposes in the Czech Republic during 1975–2000. We linked these records to the national cancer registry, revealing a total of 485 cancer cases. Subjects were classified according to the percentiles of CA distribution within each laboratory as low (0–33rd percentile), medium (34–66th percentile), and high (66–100th percentile). Subjects were further classified by occupational exposure and by subclass of CA. We found a significant association between the overall cancer incidence and the presence of chromosome-type aberrations [relative risk (RR) for high vs. low CA level = 1.24; 95% confidence interval (CI), 1.03–1.50] but not chromatid-type aberrations. Stomach cancer showed a strong association with frequency of total CA (RR = 7.79; 95% CI, 1.01–60.0). The predictivity of CA observed in subjects exposed to various classes of carcinogens did not significantly differ from the group of nonexposed subjects. This study contributes to validation of CA as a predictive marker of cancer risk, in particular, of stomach cancer; the association between CA frequency and cancer risk might be limited to chromosome-type aberrations.

The frequency of chromosomal aberrations (CAs) in human peripheral blood lymphocytes (PBLs) measured with the conventional cytogenetic assay in metaphase cells has routinely been used for several decades as a tool to monitor occupational and environmental exposures to genotoxic carcinogens. There is ample evidence of the value of this biomarker for the identification of occupational and environmental hazards (Albertini et al. 2000; Bonassi et al. 2005; Carrano and Natarajan 1988; Rossner et al. 1995; Šrám and Binkova 2000; Waters et al. 1999). However, the concept of chromosomal damage as a biomarker of early carcinogenic effects rests on the evidence of an association between biomarker frequency and cancer risk, in addition to that of an association between biomarker and exposure to genotoxic agents.

The hypothesis of a positive association between the frequency of CAs in PBLs and the risk of cancer at different sites has been supported—besides theoretical considerations (Cheng and Loeb 1993; Mitelman 2000; Sorsa et al. 1992; Yunis 1983)—by numerous clinical observations, in particular, of patients suffering from hereditary chromosome breakage syndromes (Mathur et al. 2000) and several other precancerous conditions such as preleukemic states of adult T-cell leukemia (Nishino 1988), dysplastic nevus syndrome (Caporaso et al. 1987), or nevoid basal-cell syndrome (Shafei-Benaissa et al. 1998). Case-series and case–control studies have reported a significant increase in the frequency of aberrant cells in untreated cancer patients (Abarbanel et al. 1991; Barrios et al. 1988, 1991; Dave et al. 1995; Dhillon and Dhillon 1998; Dhillon et al. 1996; Gebhart et al. 1993; Trivedi et al. 1998), but these studies have been subject to criticism because of small sample sizes and not accounting for the inherent reverse causality bias, that is, when the biomarker may be affected by the disease.

More conclusive evidence on the association between CA and cancer comes from prospective cohort studies (Bonassi et al. 2004). An increased risk of cancer incidence was observed in individuals classified as having high CA frequency in a Nordic cohort (Hagmar et al. 1994, 1998), in an Italian cohort (Bonassi et al. 1995), and, limited to chromosome-type aberrations (CSAs) in a nested case–control study carried out in Taiwan (Liou et al. 1999). In a case–control study nested within the joint Nordic and Italian cohorts, the association between CA frequency and risk of cancer was not modified by sex, age, cigarette smoking, occupational exposure, or time since the cytogenetic assay (Bonassi et al. 2000).

In the Czech Republic, the evaluation of CA frequency in PBLs has been included since 1975 in regular medical checkups of workers exposed to selected occupational hazards, making it feasible to identify a cohort of individuals for prospective follow-up for cancer in order to confirm the results of the studies from Nordic countries, Italy, and Taiwan. The large number of individuals with CA measurements and the detailed cytogenetic records allowed us to test the hypothesis that specific cytogenetic end points may be linked to the incidence of cancer at specific anatomical sites, thus expanding our preliminary results, in particular, those concerning a group of miners exposed to radon (Smerhovsky et al. 2001, 2002).

## Materials and Methods

### Study population.

The study was approved by the ethical committee of the National Institute of Public Health (Prague) and consisted of subjects examined in the period between 7 May 1975 and 7 April 2000. An overall number of 22,427 cytogenetic analyses were obtained from 15 collaborating cytogenetic laboratories. We excluded 1,387 (6.2%) results with either incomplete data on subjects’ identification or based on fewer than 100 metaphases; we also excluded 257 assays corresponding to 117 subjects with a diagnosis of cancer before the date of the first assay. Therefore, 11,991 subjects and 20,783 results were available for analysis. Many of the subjects included in the study were repeatedly examined [3,305 subjects (27.6%) underwent two or more analyses]. However, because subjects in the highest CA level at first analysis were more frequently reexamined, in all subsequent analyses we used the result of the first cytogenetic assay for all subjects. Finally, we restricted all of the analyses to subjects having 100 metaphases evaluated to avoid those subjects with ad hoc ascertainment often due to unusual exposures, leaving 11,834 subjects in the study, which contributed 113,967 person-years to the total follow-up time.

Most subjects (*n* = 9,776, 82.6%) underwent cytogenetic testing because of occupational exposure to known or suspected genotoxic agents. A smaller group (*n* = 1,913) included subjects who were involved as controls in biomonitoring studies. Subjects were stratified into five groups according to most important occupational exposures. For 1.2% (*n* = 145) of the participants, we were not able to establish the reason for the cytogenetic analysis, and these subjects were added to the group classified as “other exposures.”

### Cytogenetic analysis.

The cytogenetic analysis was carried out in 15 cytogenetic laboratories of the Czech Public Health Service. All laboratories used the same protocol for the whole study period. We used the conventional Hungerford method on short-term cultures for 50 hr, with all cells being in the first division. Peripheral blood was collected by venopuncture into heparinized tubes, and whole blood cultures were established within 24 hr from the blood collection. Tubes with heparinized blood were kept at 4–8°C until use. Cultures were set up in RPMI 1640 medium supplemented with 20% calf serum and 1% phytohemagglutinin. Two hours before harvesting, colchicin was added. Cells were collected by centrifugation, resuspended in a prewarmed hypotonic solution (0.075 M KCl) for 20 min, and fixed in acetic acid/methanol (1:3, vol/vol) on slides. These were air dried and stained with 5% Giemsa solution (pH 6.8). Slides from each culture were numbered and blindly scored. At least 100 well-spread metaphases with 46 ± 1 centromeres were examined (Bavorova et al. 1989; Rossner et al. 1998). Total CAs were subclassified as CSAs (including chromosome-type breaks, ring chromosomes, marker chromosomes, and dicentrics) and chromatid-type aberrations (CTAs; including chromatid-type breaks and chromatid exchanges) (Hagmar et al. 2004). Gaps were not scored as aberrations.

### Cancer incidence and mortality.

Information on the incidence of cancer and cause-specific mortality of members of the cohort was obtained from the National Cancer Registry for the period 1 May 1975 through 31 May 2001 (end of follow-up) and coded according to the *International Classification of Diseases*, 9th Revision [ICD-9; World Health Organization (WHO) 1975]. The link between our records and the database of the cancer registry was based on unique personal identification numbers. In the case of uncertainty, we checked the relevant code in records kept by employers. The overall number of cancer cases was 485, including 32 cases of carcinoma *in situ*. A total of 257 subjects were excluded because of the onset of cancer before the date of assay.

### Statistical methods.

To standardize for interlaboratory variability, subjects were classified according to the percentiles of CA distribution within each laboratory as low (0–33rd percentile), medium (34–66th percentile), or high (67–100th percentile). Given the low absolute frequency of CTAs and CSAs, subjects were classified according to presence or absence of these aberrations.

We used the Cox regression analysis (Cox and Oakes 1990) to model the association between cancer incidence and CA frequency. All models included age at first test and sex; time from the first test was included as a time-dependent variable. In addition, tobacco smoking (yes/no/ex-smoker) and occupational exposure (recoded in six classes) were included as potential confounders. Routine diagnostic tests did not detect any substantial violation of underlying assumptions of the Cox regression. We used SPSS for Windows (SPSS Inc., Chicago, IL, USA) and Stata statistical software (Stata Corporation, College Station, TX, USA) for all analyses.

## Results

The major descriptive characteristics of the cohort are presented in Table 1, stratified according to occupational exposure. Total CA frequency, CTAs, and CSAs were significantly higher (*p* < 0.001) in the group occupationally exposed to ionizing radiation compared with unexposed referents.

A small but not statistically significant increase in relative risk (RR) was observed for total cancer incidence in subjects with medium and high levels of CAs when compared with the low levels, whereas a significant 24% increase in RR [95% confidence interval (CI), 3–50%] was found in subjects bearing one or more CSAs (Table 2).

In the analysis of specific cancer sites, we found a significant association between a high level of CAs and cancer of digestive organs (RR = 1.86; 95% CI, 1.05–3.28), particularly for stomach cancer, with an RR of 7.79 (95% CI, 1.01–60.03). A significant increase in the RR of cancers of other and unspecified sites was found in subjects with CSAs.

In the groups defined by occupational exposure, we did not observe significant associations between CA frequency and RR of all cancers (Table 3). The only significant finding was the increased risk of all cancers among workers exposed to polyclic aromatic hydrocarbons with CSAs (RR = 1.56; 95% CI, 1.01–2.41).

The cross-tabulation of occupational exposures with selected cancer sites produced significant findings only for cancers of digestive organs. The small number of cases limited this analysis, although a stronger association between chromosomal damage and cancers of digestive organs was evident among workers exposed to ionizing radiation, where no cases were found in the lowest CA frequency group, whereas three and nine were found in the medium and high groups, respectively. This skewed distribution of cases made it impossible to estimate RRs, but the test of linear trend was highly significant (*p* < 0.01).

In order to evaluate if the inclusion of tumors *in situ* (*n* = 32) or nonmelanoma skin cancers (*n* = 70) may have affected the association between cancer risk and chromosome damage because of detection bias, we fitted models with and without these cases. The model without these cases showed a little stronger association that was still not significant (RR_medium_ = 1.27; 95% CI, 0.97–1.67; RR_high_ = 1.22; 95% CI, 0.93–1.59).

## Discussion

The role of some chemicals and ionizing radiation in inducing DNA double-strand breaks that, if not repaired, are transformed into CAs during cell division is well established (Bryant 1998; Natarajan 1993; Obe et al. 2002; Palitti 1998; Savage 1998). Measuring the frequency of chromosomal damage in humans exposed to occupational and environmental clastogens has been a priority in public health studies for decades, and an increased level of CAs in population groups is currently interpreted as evidence of genotoxic exposure and early biologic effects on DNA (Albertini et al. 2000; Šrám 1981; Šrám et al. 1983; Waters et al. 1999). However, before using CAs as a marker of cancer risk, it is essential to establish not only the presence of an association with exposure but also the link with cancer occurrence (WHO 2001).

The results from this study contribute important evidence on CAs as a predictive cancer biomarker. One strength of these findings is the homogeneity of cytogenetic protocols in the laboratories included in the study, which should have reduced the misclassification due to technical variability. Furthermore, the size of this cohort, which is more than double the size of the combined Nordic–Italian cohorts (Hagmar et al. 1998), allows the analysis of specific cancer sites, the study of interaction with occupational exposures, and the evaluation of subclasses of CAs.

The association between the total frequency of CAs and all cancer incidence was quantitatively lower than that reported in previous studies (Hagmar et al. 1998, 2004), at least regarding the risk for those subjects in the highest tertile of the distribution of CA frequency. A possible explanation of this finding is the implementation of preventive interventions after the detection of a subject with a high CA level. In the Czech Republic, CA surveying was part of a systematic effort to provide an early detection of occupational damages, and subjects with a CA frequency of ≥4% were included in a program that was intended to reduce the risks for these individuals. An alternative explanation is that the association between CA frequency and cancer risk is weaker than previously considered.

Our results on the predictivity of CA subclasses support early data from Taiwan (Liou et al. 1999) because a significant increase of incidence is described only for CSAs and not for CTAs. These findings are in agreement with the evidence that a double-strand break—which is a consolidated early event of carcino-genesis—is the primary lesion for CSAs, and that agents that produce double-strand breaks, such as ionizing radiation and radiomimetic clastogenic chemicals, create CSAs (Pfeiffer et al. 2000). Such a difference was not detected in the combined analysis of the Nordic and Italian cohorts (Hagmar et al. 2004).

The association between CA frequency and risk of specific cancers did not reveal a great variability, and positive associations might have been generated by multiple comparisons; however, the increase in cancers of digestive organs, and most notably stomach cancer, is a potentially important observation-that requires confirmation.

The presence of interaction between exposure to carcinogens and the predictivity of CAs has been another issue largely debated in the literature. The presence of a stronger association between CA frequency and risk of cancer in radon-exposed workers than in other workers or controls, which has been already reported (Smerhovsky et al. 2002), is not consistent with the findings of the Nordic and Italian cohorts, in which the association between increased CA frequency and cancer risk appeared to be independent from exposure to carcinogens or smoking habit (Bonassi et al. 2000). The findings from the present study were not conclusive in this direction because the predictivity of CA frequency observed in subjects exposed to various classes of carcinogens did not significantly differ from the group of nonexposed subjects. However, when the group of digestive cancers was cross-tabulated by occupational exposure, a significant association was seen only in the group of workers exposed to ionizing radiation. To better disentangle the interaction between radiation, CAs, and cancer, we further broke down CA subclasses in chromosome breaks and exchanges, and interestingly, the events mostly associated with digestive cancer incidence were exchanges, both chromatid exchanges (*p* < 0.01) and chromosome exchanges (*p* < 0.05).

In conclusion, this study confirms previous reports of an association between the extent of chromosomal damage and the risk of cancer. In contrast to most previous reports, this association appeared to be limited to the presence of CSAs, and the magnitude of the excess risk might be lower than previously described. An original result of this analysis concerns the presence of a stronger association between CA frequency and cancers of the digestive tract. Also, the higher risks found in the group exposed to ionizing radiation is a peculiar finding of this cohort and deserves a deeper insight.

Furthermore, the possibility that the implementation of occupational preventive programs focused on workers with high CA frequency might have modified their risk of cancer is a plausible explanation of these results, and it will be further evaluated with ad hoc studies, reconstructing occupational lives of subjects with the highest frequency of CA at their first cytogenetic analysis.

## Figures and Tables

**Table 1:** Distribution of subjects in the cohort by age at first test, sex, duration of follow-up, cancer frequency, and frequency of CAs.

						Median (25th–75th percentiles)		
Occupational exposure	No. of subjects	Mean age at first test (years)	Sex (% males)	Mean years since test	Cancer cases (%)	CAs	CTAs	CSAs
Ionizing radiation	676	40.3	70.4	11.6	57 (8.4)	2.5 (1.5–4.0)	2.0 (1.0–3.0)	1.0 (0.0–2.0)
Cytostatic drugs	2,150	36.5	12.8	7.4	61 (2.8)	2.0 (1.0–3.0)	1.3 (1.0–2.0)	0.0 (0.0–1.0)
Polyclic aromatic hydrocarbons	2,241	39.4	86.1	10.1	108 (4.8)	2.0 (1.0–3.5)	1.5 (1.0–2.4)	1.0 (0.0–1.0)
Aromatic amines	851	40.3	57.8	9.0	28 (3.3)	2.0 (1.3–3.0)	1.8 (1.0–3.0)	0.6 (0.0–1.0)
Other exposures	4,031	38.0	58.7	10.7	170 (4.2)	2.0 (1.0–3.0)	1.0 (1.0–2.0)	0.5 (0.0–1.0)
Nonexposed	1,913	36.2	55.2	8.8	61 (3.2)	2.0 (1.0–3.0)	1.0 (0.5–2.0)	0.0 (0.0–1.0)
Total cohort	11,862	38.0	55.6	9.6	485 (4.1)	2.0 (1.0–3.0)	1.0 (1.0–2.0)	0.5 (0.0–1.0)

**Table 2:** Results from the multivariate Cox regression analysis of CA frequency (total and by subclass) and cancer incidence.

		Incident cases (by tertile of CA distribution)			Total CAs*^a^* (by tertile of CA distribution)		RR_≥1_ (95% CI)	
Cancer site	ICD-9 code	Low	Medium	High	RR_medium_ (95% CI)	RR_high_ (95% CI)	CTAS*^b^* (≥1 vs. 0)	CSAs*^b^* (≥1 vs. 0)
Lip, oral cavity, and pharynx	140–149	4	1	9	0.23 (0.03–2.06)	1.89 (0.55–6.50)	1.61 (0.36–7.33)	1.98 (0.61–6.42)
Digestive organs	150–159	16	31	48	1.47 (0.80–2.70)	1.86 (1.05–3.28)	1.20 (0.69–2.09)	1.46 (0.94–2.27)
Stomach	151	1	3	12	2.25 (0.23–21.66)	7.79 (1.01–60.03)	1.43 (0.32–6.30)	2.79 (0.79–9.83)
Colon, rectum	153–154	13	24	22	1.41 (0.72–2.79)	0.94 (0.47–1.89)	1.12 (0.56–2.22)	1.16 (0.68–1.99)
Respiratory and intrathoracic organs	160–165	19	22	38	0.87 (0.47–1.61)	1.02 (0.58–1.80)	0.86 (0.49–1.50)	1.26 (0.78–2.03)
Trachea, bronchus, and lung	162	18	21	34	0.87 (0.46–1.64)	0.96 (0.54–1.74)	0.84 (0.48–1.50)	1.27 (0.77–2.10)
Bone, connective tissue, skin, and breast	170–175	31	51	46	1.29 (0.82–2.01)	1.08 (0.68–1.73)	1.19 (0.75–1.90)	0.88 (0.62–1.26)
Skin (nonmelanoma)	173	20	25	25	0.97 (0.54–1.75)	0.89 (0.49–1.63)	1.01 (0.56–1.83)	0.81 (0.50–1.30)
Breast	174	9	19	11	1.60 (0.72–3.55)	0.97 (0.40–2.36)	0.97 (0.44–2.13)	1.05 (0.54–2.05)
Genitourinary organs	179–189	33	45	40	1.05 (0.67–1.66)	0.82 (0.51–1.32)	0.93 (0.59–1.46)	1.17 (0.80–1.71)
Uterus	179–182	15	19	16	1.02 (0.52–2.01)	0.94 (0.46–1.92)	0.89 (0.45–1.74)	1.32 (0.74–2.34)
Prostate	185	3	2	7	0.49 (0.08–3.10)	1.23 (0.29–5.28)	0.81 (0.21–3.16)	0.70 (0.20–2.41)
Bladder	188	6	3	4	0.38 (0.09–1.53)	0.34 (0.09–1.27)	0.78 (0.21–2.88)	0.70 (0.23–2.11)
Other and unspecified sites	190–199	5	12	8	1.94 (0.68–5.54)	1.17 (0.38–3.64)	0.99 (0.37–2.68)	3.91 (1.33–11.54)
Lymphatic and hematopoietic tissue	200–208	5	11	10	1.70 (0.59–4.91)	1.49 (0.50–4.44)	0.66 (0.28–1.58)	1.73 (0.75–4.04)
Total cancers	140–208	113	173	199	1.17 (0.92–1.48)	1.13 (0.89–1.43)	1.02 (0.81–1.28)	1.24 (1.03–1.50)*

a Reference level: lowest tertile of CA distribution.

b Reference level: subjects with “0” CTAs or CSAs.

* *p* < 0.05.

**Table 3:** Results from the multivariate Cox regression analysis of CA frequency (total and by subclass) and total cancer incidence by occupational exposure.

		Total CAs*^a^* (by tertile of CA distribution)		RR_≥1_ (95% CI)	
Occupational exposure	No. of subjects	RR_medium_ (95% CI)	RR_high_ (95% CI)	CTAs*^b^* (≥1 vs. 0)	CSAs*^b^* (≥1 vs. 0)
Ionizing radiation	676	1.42 (0.61–3.33)	1.39 (0.61–3.16)	1.35 (0.62–2.89)	1.17 (0.65–2.10)
Cytostatic drugs	2,150	1.09 (0.57–2.07)	0.96 (0.50–1.85)	0.95 (0.50–1.79)	1.33 (0.79–2.25)
Polyclic aromatic hydrocarbons	2,241	1.27 (0.73–2.25)	1.39 (0.82–2.34)	1.20 (0.70–2.04)	1.56 (1.01–2.41)
Aromatic amines	851	1.85 (0.59–5.80)	1.29 (0.41–4.07)	1.06 (0.31–3.61)	0.84 (0.40–1.78)
Other exposures	4,031	1.04 (0.71–1.53)	0.86 (0.58–1.26)	0.83 (0.57–1.29)	1.17 (0.85–1.61)
Nonexposed	1,913	1.23 (0.68–2.22)	1.55 (0.80–3.01)	1.13 (0.63–2.03)	1.33 (0.80–2.21)
Total	11,862	1.17 (0.92–1.48)	1.13 (0.88–1.43)	1.02 (0.81–1.28)	1.24 (1.03–1.50)^*^

a Reference level: lowest tertile of CA distribution.

b Reference level: subjects with “0” CTAs or CSAs.

* *p* < 0.05.
